# *N* ^3^,*N* ^6^,2,5,7-Penta­phenyl-2,5,7-triaza­bicyclo­[2.2.1]heptane-3,6-diamine

**DOI:** 10.1107/S1600536809024416

**Published:** 2009-07-01

**Authors:** Amir Taheri, Sayed Mojtaba Moosavi

**Affiliations:** a Department of Chemistry, Imam Hossein University, Tehran, Iran

## Abstract

In the title compound, C_34_H_31_N_5_, the observed molecular geometry suggests that anomeric effects are present in terms of short C—N bond lengths and reduced pyramidality of the N atoms.

## Related literature

For the synthesis of the title compound and the structure of another 2,5,7–triaza­bicyclo­[2.2.1]heptan derivative, see: Taheri & Moosavi (2009 ▶). For its precursors, see: Kliegman & Barnes (1970 ▶); Taheri & Moosavi (2008 ▶). For general background to aza­norbornanes, see Alphen, (1933 ▶); Alvaro *et al.* (2007 ▶); Archelas & Morin (1984 ▶); Nitravati & Sikhibhushan (1939 ▶, 1941 ▶); Potts & Husain (1972 ▶); Potts *et al.* (1974 ▶); Neunhoeffer & Fruhauf (1969 ▶, 1970 ▶); Stanforth *et al.* (2002 ▶). For the syntheses of polyaza­polycyclic compounds, see: Nielsen *et al.* (1990 ▶, 1992 ▶, 1998 ▶). For the anomeric effect, see: Senderowitz *et al.* (1992 ▶); Reed & Schleyer (1988 ▶); Watson *et al.* (1990 ▶); Davies *et al.* (1992 ▶). 
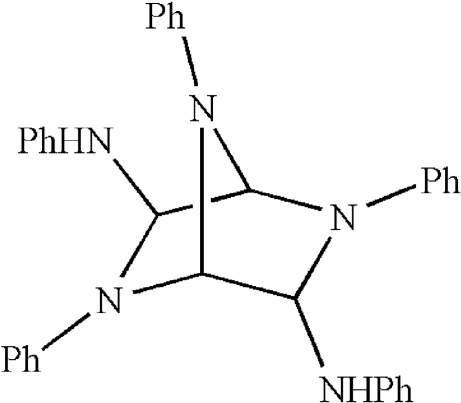


## Experimental

### 

#### Crystal data

C_34_H_31_N_5_
*M* *_r_* = 509.64
Orthorhombic, 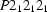

*a* = 9.7427 (4) Å
*b* = 16.4049 (7) Å
*c* = 17.0658 (7) Å
*V* = 2727.6 (2) Å^3^
*Z* = 4
Mo *K*α radiation
μ = 0.08 mm^−1^
*T* = 100 K
0.25 × 0.15 × 0.10 mm

#### Data collection

Bruker APEXII CCD area-detector diffractometer
Absorption correction: multi-scan (*SADABS*; Sheldrick, 2003 ▶) *T*_min_ = 0.981, *T*_max_ = 0.990
27681 measured reflections
3131 independent reflections
2816 reflections with *I* > 2σ(*I*)
*R*_int_ = 0.060

#### Refinement

*R*[*F* ^2^ > 2σ(*F* ^2^)] = 0.031
*wR*(*F* ^2^) = 0.072
*S* = 1.01
3131 reflections
352 parameters
H-atom parameters constrained
Δρ_max_ = 0.17 e Å^−3^
Δρ_min_ = −0.17 e Å^−3^

### 

Data collection: *APEX2* (Bruker, 2005 ▶); cell refinement: *SAINT-Plus* (Bruker, 2001 ▶); data reduction: *SAINT-Plus*; program(s) used to solve structure: *SHELXTL* (Sheldrick, 2008 ▶); program(s) used to refine structure: *SHELXTL*; molecular graphics: *SHELXTL*; software used to prepare material for publication: *SHELXTL*.

## Supplementary Material

Crystal structure: contains datablocks I, global. DOI: 10.1107/S1600536809024416/rk2152sup1.cif

Structure factors: contains datablocks I. DOI: 10.1107/S1600536809024416/rk2152Isup2.hkl

Additional supplementary materials:  crystallographic information; 3D view; checkCIF report

## Figures and Tables

**Fig. 1.**
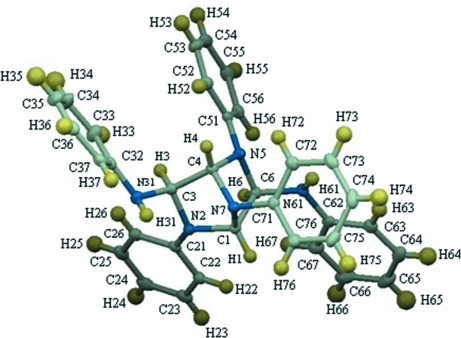
The molecular structure of I, with the atom–numbering scheme. Displacement ellipsoids are drawn at the 50% probability level. H atoms are presented as a small spheres of arbitrary radius.

## supplementary crystallographic information

### Comment

There are different kinds of polyazapolycyclic skeletons (Nielsen *et al.*, 1990) constituted of saturated rings with multiple N atoms, that can be utilized for high–density and energetic compounds syntheses (Nielsen *et al.*, 1992). In cage skeleton, 2,4,6,8,10,12–hexabenzyl–2,4,6,8,10,12–hexaazaisowurtzitane is precursor for 2,4,6,8,10,12–hexanitro–2,4,6,8,10,12–hexaazaisowurtzitane, which is highly energetic compound (Nielsen *et al.*, 1998). In norbornane skeletons, azanorbornane or azabicyclo[2.2.1]heptane (Archelas & Morin, 1984) and diazanorbornane derivatives (Alvaro *et al.*, 2007) have been synthesized and characterized so far, but triazanorbornane derivatives have seldom been reported (Nitravati & Sikhibhushan, 1939, 1941; Alphen, 1933). The syntheses and molecular structures of triazabicyclo[2.2.1]heptaneshave been presented in a few papers without using X–ray crystal structure analysis (Potts & Husain, 1972; Potts *et al.*, 1974; Neunhoeffer & Fruhauf, 1969,1970; Stanforth *et al.*, 2002).

As a part of our continuing efforts on the development of polyazapolycyclics, structural stability and synthesis of 2,5,7–triazabicyclo[2.2.1]heptan derivative (Taheri & Moosavi, 2009) *via* a catalytic reaction between aminoethane derivatives (Kliegman & Barnes, 1970; Taheri & Moosavi, 2008) and glyoxal were recently described another crystal system of the title compound without any solvent on the crystal packing in which geometric parameters for stability of the skeleton is scrutinized by study of anomeric interactions.

The molecular structure of I shown in Fig. 1 has racemic configuration, all *S*– and all *R*–configuration molecules and composed of a six–membered piperazine ring and an N atom bridging between the C1 and C4 situations, norbornane skeleton construction. Notwithstanding the presence of two NH groups, *viz.* N31 and N61, and five N atoms carrying lone–pair electrons potentially available for H–bond creation, there are not actually intra– or intermolecular N—H···N or C—H···N hydrogen bonds. As shown in the scheme, the skeleton has a good local twofold symmetry, namely through the N7 bridge and almost perpendicular to the least–squares plane of the piperazine ring. It is noteworthy that the symmetry involves not only the skeleton but also the peripheral phenyl groups, except for that attached to the bridging N7 atom.

The anomeric effect in N—C—N systems investigated extensively (Senderowitz *et al.*, 1992), occurred between a lone pair on N and an antiperiplanar σ* orbital of the adjacent C—N bond (*n*_N_→σ*_C—N_), negative hyperconjugation (Reed & Schleyer, 1988).

There are four dissimilar anomeric effects manifested by the bond distances and N–atom pyramidality on four N'—C—N" fragments or *n*_N_'→σ*_C—N_" systems. Within the N'—C—N" unit, the N'—C bond is shorter, than the C—N" bond. On the other hand, the pyramidality of N' (the sum of the three bond angles around N') is larger than that of N". These geometric parameters related to the anomeric effect are shown in Tabl. 1. Among them, the *n*_N5_→σ*_C4—N7_ system shows a distinguished anomeric interaction and the largest bond–length difference [0.029 (2)Å], which is comparable to that reported for an other crystal system (Taheri & Mossavi, 2009).

However, the pyramidality differences in the N'—C—N" units are not so indicative. The differences within the N2—C2—N7 and N7—C4—N5 systems are 16.14 (16) and 17.83 (15)°, respectively, and these are much larger, than those for the N31—C3—N2 and N61—C6—N5 groups, 2.95 and 0.21°, respectively. Furthermore, the calculated pyramidalities for atoms N31 and N61 are not accurate because they include H atoms, whose positions were determined from adifference Fourier synthesis. Thus, the anomeric effect on the pyramidality is not clear in this molecule. It could be that, the anomeric effect on the angle is buried among the steric effects caused by the crowding of the substituent groups, which would strongly affect the molecular structure.

Reflecting the local twofold symmetry, the corresponding N atoms in this symmetric skeleton (N2 and N5, N31 and N61) have nearly the same pyramidality. The pyramidality angle of N7 [330.71 (19)°] is rather small, and the attached phenyl group is inclined from the local twofold axis in the direction of atoms N2, C3 and N31. Corresponding to this inclination, the C72—C71—N7 angle [122.79 (17)°] is distorted from theideal value of 120°, which is attributable to the short contact between the voluminous phenyl ring and the skeleton. For example, the H76···H1 separation (atom C1 is the bridgehead) is only 2.281Å . The same distortion is seen in another norbornane derivative [122.5 (4)°; Watson *et al.*, 1990] for the phenyl ring on the bridging N7 atom.

The angle at the bridging N atom, C1—N7—C4, is 94.11 (13)°. Although this bridge angle is comparable to those reported for norbornane and diazanorbornane (Davies *et al.*, 1992), it still indicates the presence of ring strain.

### Experimental

Aqueous glyoxal (40% *v*/*v*, 1.15 ml, 0.01 mol) was added dropwise to a stirred solution of 1,1',2,2'–tetrakis(phenylamino)ethane (3.94 g, 0.01 mol) in ethanol (50 ml). The solution temperature was kept at 273 K during the reaction. The mixture was put aside for 24 h at a temperature of 278–283 K. The resulting white precipitate was filtered off and washed with cold ethanol to give 2.53 g (55% yield) of I (m.p. 428 K).

^1^H NMR (CDCl_3_): ^1^H 6.59–7.30 (m, 25H, CH*_Ar_*), 5.69 (s, 2H, CH), 4.93 (d, 2H, *J* = 10 Hz, CH), 3.69 (d, 2H, *J* = 10 Hz, NH). Addition of D_2_O to the NMR sample caused the NH signals to disappeared and the CH doublet quickly converted to a singlet.

^13^C NMR (CDCl_3_): ^13^C 144.8, 144.2, 143.6,129.3, 129.7, 122.1, 119.4, 118.7, 117.4, 113.7, 113.2 (CH*_Ar_*), 76.0 (CH), 72.4 (CH).

### Refinement

The H atoms of the NH–groups were located in the difference Fourier map and refined in rigid model with fixed *U*_iso_(H) = 1.2*U*_eq_(N) parameters. The H(C) atoms were placed in calculated positions and refined in riding model with fixed *U*_iso_(H) = 1.2*U*_eq_(C) parameters. Friedel opposites were merged

### Crystal data

**Table:**

C_34_H_31_N_5_	*F*(000) = 1080
*M**_r_* = 509.64	*D*_x_ = 1.241 Mg m^−^^3^
Orthorhombic, *P*2_1_2_1_2_1_	Mo *K*α radiation, λ = 0.71073 Å
Hall symbol: P 2ac 2ab	Cell parameters from 4705 reflections
*a* = 9.7427 (4) Å	θ = 2.4–22.0°
*b* = 16.4049 (7) Å	µ = 0.08 mm^−^^1^
*c* = 17.0658 (7) Å	*T* = 100 K
*V* = 2727.6 (2) Å^3^	Prism, colourless
*Z* = 4	0.25 × 0.15 × 0.10 mm

### Data collection

**Table:**

Bruker SMART APEXII CCD area-detector diffractometer	3131 independent reflections
Radiation source: Fine–focus sealed tube	2816 reflections with *I* > 2σ(*I*)
Graphite	*R*_int_ = 0.060
φ and ω scans	θ_max_ = 26.4°, θ_min_ = 1.7°
Absorption correction: multi-scan (*SADABS*; Sheldrick, 2003)	*h* = −12→12
*T*_min_ = 0.981, *T*_max_ = 0.990	*k* = −20→20
27681 measured reflections	*l* = −21→21

### Refinement

**Table:**

Refinement on *F*^2^	Secondary atom site location: difference Fourier map
Least-squares matrix: Full	Hydrogen site location: inferred from neighbouring sites
*R*[*F*^2^ > 2σ(*F*^2^)] = 0.031	H-atom parameters constrained
*wR*(*F*^2^) = 0.072	*w* = 1/[σ^2^(*F*_o_^2^) + (0.0373*P*)^2^ + 0.373*P*] where *P* = (*F*_o_^2^ + 2*F*_c_^2^)/3
*S* = 1.01	(Δ/σ)_max_ < 0.001
3131 reflections	Δρ_max_ = 0.17 e Å^−^^3^
352 parameters	Δρ_min_ = −0.17 e Å^−^^3^
0 restraints	Absolute structure: 2419 Friedel pairs were merged
Primary atom site location: structure-invariant direct methods	

### Special details

**Table:**

Geometry. All s.u.'s (except the s.u. in the dihedral angle between two l.s. planes) are estimated using the full covariance matrix. The cell s.u.'s are taken into account individually in the estimation of s.u.'s in distances, angles and torsion angles; correlations between s.u.'s in cell parameters are only used when they are defined by crystal symmetry. An approximate (isotropic) treatment of cell s.u.'s is used for estimating s.u.'s involving l.s. planes.
Refinement. Refinement of *F*^2^ against ALL reflections. The weighted *R*–factor *wR* and goodness of fit *S* are based on *F*^2^, conventional *R*–factors *R* are based on *F*, with *F* set to zero for negative *F*^2^. The threshold expression of *F*^2^ > σ(*F*^2^) is used only for calculating *R*–factors(gt) *etc*. and is not relevant to the choice of reflections for refinement. *R*–factors based on *F*^2^ are statistically about twice as large as those based on *F*, and *R*–factors based on ALL data will be even larger.

### Fractional atomic coordinates and isotropic or equivalent isotropic displacement parameters (Å^2^)

**Table:**

	*x*	*y*	*z*	*U*_iso_*/*U*_eq_	
C1	0.66064 (19)	0.61819 (11)	0.23890 (10)	0.0161 (4)	
H1A	0.6227	0.6561	0.2793	0.019*	
N2	0.70778 (16)	0.53934 (9)	0.26954 (8)	0.0163 (3)	
C3	0.71251 (19)	0.48417 (10)	0.20155 (10)	0.0164 (4)	
H3A	0.8095	0.4679	0.1904	0.020*	
C4	0.65972 (18)	0.54197 (11)	0.13717 (10)	0.0159 (4)	
H4A	0.6216	0.5137	0.0900	0.019*	
N5	0.77088 (16)	0.60077 (9)	0.11985 (8)	0.0163 (3)	
C6	0.77956 (19)	0.65473 (11)	0.18816 (10)	0.0164 (4)	
H6A	0.8698	0.6478	0.2153	0.020*	
N7	0.56002 (15)	0.58946 (9)	0.18081 (8)	0.0158 (3)	
C21	0.80912 (18)	0.53697 (11)	0.32784 (10)	0.0165 (4)	
C22	0.82474 (19)	0.60204 (11)	0.38008 (10)	0.0184 (4)	
H22A	0.7685	0.6490	0.3746	0.022*	
C23	0.9213 (2)	0.59879 (12)	0.43974 (11)	0.0228 (4)	
H23A	0.9299	0.6433	0.4750	0.027*	
C24	1.0055 (2)	0.53119 (13)	0.44833 (11)	0.0246 (4)	
H24A	1.0723	0.5293	0.4889	0.030*	
C25	0.9907 (2)	0.46637 (13)	0.39682 (11)	0.0240 (4)	
H25A	1.0482	0.4199	0.4021	0.029*	
C26	0.89300 (19)	0.46840 (12)	0.33759 (11)	0.0209 (4)	
H26A	0.8830	0.4230	0.3035	0.025*	
N31	0.62863 (16)	0.41223 (9)	0.21507 (9)	0.0181 (3)	
H31N	0.5424	0.4272	0.2275	0.022*	
C32	0.6379 (2)	0.34644 (11)	0.16289 (10)	0.0180 (4)	
C33	0.7651 (2)	0.31662 (12)	0.13812 (12)	0.0266 (4)	
H33A	0.8470	0.3432	0.1541	0.032*	
C34	0.7723 (2)	0.24833 (13)	0.09027 (13)	0.0333 (5)	
H34A	0.8595	0.2287	0.0739	0.040*	
C35	0.6546 (2)	0.20845 (12)	0.06601 (13)	0.0316 (5)	
H35A	0.6604	0.1617	0.0333	0.038*	
C36	0.5284 (2)	0.23776 (12)	0.09010 (11)	0.0259 (5)	
H36A	0.4469	0.2108	0.0739	0.031*	
C37	0.5193 (2)	0.30628 (11)	0.13780 (10)	0.0207 (4)	
H37A	0.4318	0.3259	0.1535	0.025*	
C51	0.88738 (19)	0.57525 (11)	0.07785 (10)	0.0157 (4)	
C52	0.8730 (2)	0.51490 (11)	0.02028 (11)	0.0204 (4)	
H52A	0.7861	0.4902	0.0118	0.024*	
C53	0.9849 (2)	0.49093 (12)	−0.02443 (11)	0.0236 (4)	
H53A	0.9735	0.4505	−0.0637	0.028*	
C54	1.1129 (2)	0.52537 (12)	−0.01237 (11)	0.0231 (4)	
H54A	1.1898	0.5078	−0.0421	0.028*	
C55	1.1272 (2)	0.58591 (12)	0.04381 (11)	0.0227 (4)	
H55A	1.2143	0.6106	0.0517	0.027*	
C56	1.0161 (2)	0.61088 (11)	0.08871 (11)	0.0194 (4)	
H56A	1.0277	0.6524	0.1270	0.023*	
N61	0.76301 (16)	0.73814 (9)	0.16149 (9)	0.0179 (3)	
H61N	0.6972	0.7403	0.1234	0.021*	
C62	0.76841 (19)	0.80551 (11)	0.21117 (10)	0.0173 (4)	
C63	0.6967 (2)	0.87592 (11)	0.18892 (12)	0.0217 (4)	
H63A	0.6411	0.8750	0.1432	0.026*	
C64	0.7058 (2)	0.94660 (12)	0.23260 (13)	0.0286 (5)	
H64A	0.6594	0.9944	0.2156	0.034*	
C65	0.7825 (2)	0.94832 (13)	0.30131 (13)	0.0328 (5)	
H65A	0.7871	0.9965	0.3320	0.039*	
C66	0.8519 (2)	0.87877 (13)	0.32424 (13)	0.0302 (5)	
H66A	0.9041	0.8796	0.3712	0.036*	
C67	0.8468 (2)	0.80757 (12)	0.27986 (11)	0.0221 (4)	
H67A	0.8963	0.7606	0.2961	0.027*	
C71	0.48078 (18)	0.65048 (11)	0.14169 (11)	0.0168 (4)	
C72	0.49051 (19)	0.66496 (11)	0.06118 (11)	0.0196 (4)	
H72A	0.5510	0.6332	0.0299	0.024*	
C73	0.4118 (2)	0.72570 (12)	0.02695 (12)	0.0236 (4)	
H73A	0.4190	0.7353	−0.0278	0.028*	
C74	0.3232 (2)	0.77245 (12)	0.07143 (13)	0.0276 (5)	
H74A	0.2707	0.8146	0.0477	0.033*	
C75	0.3116 (2)	0.75718 (12)	0.15116 (13)	0.0273 (5)	
H75A	0.2506	0.7890	0.1820	0.033*	
C76	0.3880 (2)	0.69600 (11)	0.18629 (11)	0.0216 (4)	
H76A	0.3773	0.6850	0.2406	0.026*	

### Atomic displacement parameters (Å^2^)

**Table:**

	*U*^11^	*U*^22^	*U*^33^	*U*^12^	*U*^13^	*U*^23^
C1	0.0168 (9)	0.0159 (8)	0.0157 (8)	0.0015 (7)	−0.0011 (7)	−0.0009 (7)
N2	0.0196 (8)	0.0148 (7)	0.0146 (7)	0.0016 (6)	−0.0009 (6)	−0.0003 (6)
C3	0.0168 (9)	0.0161 (8)	0.0164 (8)	−0.0004 (7)	0.0015 (7)	−0.0008 (7)
C4	0.0150 (8)	0.0169 (9)	0.0157 (8)	−0.0011 (7)	−0.0001 (7)	−0.0002 (7)
N5	0.0177 (8)	0.0163 (7)	0.0149 (7)	−0.0015 (6)	0.0002 (6)	−0.0020 (6)
C6	0.0182 (9)	0.0159 (8)	0.0152 (8)	0.0003 (7)	−0.0011 (8)	0.0003 (7)
N7	0.0170 (7)	0.0174 (7)	0.0131 (7)	0.0011 (6)	−0.0001 (6)	−0.0009 (6)
C21	0.0157 (9)	0.0201 (9)	0.0137 (8)	−0.0013 (7)	0.0018 (7)	0.0031 (7)
C22	0.0199 (9)	0.0190 (9)	0.0163 (9)	−0.0019 (8)	0.0004 (7)	0.0023 (8)
C23	0.0219 (10)	0.0303 (11)	0.0163 (9)	−0.0072 (9)	0.0017 (8)	0.0017 (8)
C24	0.0162 (9)	0.0405 (11)	0.0172 (9)	−0.0028 (9)	−0.0010 (8)	0.0060 (9)
C25	0.0178 (9)	0.0317 (11)	0.0224 (9)	0.0038 (9)	0.0027 (8)	0.0079 (9)
C26	0.0217 (9)	0.0223 (10)	0.0187 (9)	0.0017 (8)	0.0026 (8)	0.0016 (8)
N31	0.0170 (8)	0.0155 (7)	0.0219 (8)	−0.0007 (7)	0.0038 (7)	−0.0006 (6)
C32	0.0237 (10)	0.0141 (8)	0.0163 (8)	−0.0017 (8)	0.0013 (8)	0.0035 (7)
C33	0.0218 (10)	0.0210 (9)	0.0370 (11)	−0.0018 (8)	0.0028 (9)	−0.0053 (9)
C34	0.0308 (11)	0.0253 (10)	0.0439 (13)	0.0029 (10)	0.0108 (11)	−0.0081 (10)
C35	0.0433 (13)	0.0203 (10)	0.0313 (11)	−0.0041 (10)	0.0087 (10)	−0.0062 (9)
C36	0.0334 (11)	0.0235 (10)	0.0209 (10)	−0.0091 (9)	−0.0009 (9)	−0.0001 (8)
C37	0.0240 (10)	0.0196 (9)	0.0186 (9)	−0.0029 (8)	0.0036 (8)	0.0035 (8)
C51	0.0172 (9)	0.0157 (8)	0.0143 (8)	0.0025 (7)	0.0014 (7)	0.0039 (7)
C52	0.0184 (9)	0.0218 (9)	0.0209 (9)	−0.0019 (8)	0.0011 (8)	−0.0005 (8)
C53	0.0257 (10)	0.0223 (10)	0.0229 (9)	0.0034 (8)	0.0028 (9)	−0.0045 (8)
C54	0.0200 (10)	0.0270 (10)	0.0224 (9)	0.0075 (9)	0.0053 (8)	0.0046 (8)
C55	0.0150 (9)	0.0261 (10)	0.0269 (10)	−0.0008 (8)	−0.0016 (8)	0.0070 (8)
C56	0.0211 (9)	0.0180 (9)	0.0189 (9)	−0.0010 (8)	−0.0029 (8)	0.0025 (8)
N61	0.0227 (8)	0.0167 (7)	0.0143 (7)	−0.0021 (7)	−0.0032 (7)	0.0000 (6)
C62	0.0169 (9)	0.0172 (9)	0.0177 (8)	−0.0048 (8)	0.0051 (7)	−0.0014 (7)
C63	0.0199 (10)	0.0218 (9)	0.0236 (10)	−0.0031 (8)	0.0045 (8)	0.0014 (8)
C64	0.0281 (11)	0.0193 (10)	0.0386 (12)	−0.0029 (9)	0.0112 (10)	−0.0005 (9)
C65	0.0328 (12)	0.0255 (11)	0.0401 (12)	−0.0085 (9)	0.0115 (11)	−0.0153 (10)
C66	0.0249 (11)	0.0385 (12)	0.0272 (10)	−0.0102 (10)	0.0017 (9)	−0.0116 (9)
C67	0.0189 (9)	0.0261 (10)	0.0213 (9)	−0.0031 (8)	−0.0005 (8)	−0.0029 (8)
C71	0.0153 (9)	0.0155 (9)	0.0196 (9)	−0.0025 (7)	−0.0031 (7)	−0.0001 (7)
C72	0.0161 (9)	0.0218 (9)	0.0210 (9)	−0.0036 (8)	−0.0015 (8)	0.0004 (8)
C73	0.0246 (10)	0.0251 (10)	0.0213 (10)	−0.0080 (8)	−0.0056 (9)	0.0051 (8)
C74	0.0291 (11)	0.0192 (10)	0.0346 (12)	0.0003 (9)	−0.0126 (10)	0.0030 (9)
C75	0.0272 (11)	0.0211 (10)	0.0335 (11)	0.0069 (8)	−0.0070 (9)	−0.0081 (9)
C76	0.0245 (10)	0.0219 (10)	0.0184 (9)	0.0015 (8)	−0.0033 (8)	−0.0018 (8)

### Geometric parameters (Å, °)

**Table:**

C1—N2	1.469 (2)	C36—C37	1.391 (3)
C1—N7	1.472 (2)	C36—H36A	0.9500
C1—C6	1.566 (2)	C37—H37A	0.9500
C1—H1A	1.0000	C51—C56	1.396 (3)
N2—C21	1.402 (2)	C51—C52	1.402 (3)
N2—C3	1.472 (2)	C52—C53	1.387 (3)
C3—N31	1.454 (2)	C52—H52A	0.9500
C3—C4	1.540 (2)	C53—C54	1.385 (3)
C3—H3A	1.0000	C53—H53A	0.9500
C4—N7	1.451 (2)	C54—C55	1.388 (3)
C4—N5	1.480 (2)	C54—H54A	0.9500
C4—H4A	1.0000	C55—C56	1.388 (3)
N5—C51	1.406 (2)	C55—H55A	0.9500
N5—C6	1.466 (2)	C56—H56A	0.9500
C6—N61	1.451 (2)	N61—C62	1.394 (2)
C6—H6A	1.0000	N61—H61N	0.9140
N7—C71	1.430 (2)	C62—C67	1.399 (3)
C21—C22	1.399 (2)	C62—C63	1.402 (3)
C21—C26	1.400 (3)	C63—C64	1.381 (3)
C22—C23	1.387 (3)	C63—H63A	0.9500
C22—H22A	0.9500	C64—C65	1.390 (3)
C23—C24	1.387 (3)	C64—H64A	0.9500
C23—H23A	0.9500	C65—C66	1.383 (3)
C24—C25	1.387 (3)	C65—H65A	0.9500
C24—H24A	0.9500	C66—C67	1.393 (3)
C25—C26	1.389 (3)	C66—H66A	0.9500
C25—H25A	0.9500	C67—H67A	0.9500
C26—H26A	0.9500	C71—C72	1.397 (2)
N31—C32	1.402 (2)	C71—C76	1.398 (3)
N31—H31N	0.9006	C72—C73	1.386 (3)
C32—C37	1.398 (3)	C72—H72A	0.9500
C32—C33	1.398 (3)	C73—C74	1.382 (3)
C33—C34	1.388 (3)	C73—H73A	0.9500
C33—H33A	0.9500	C74—C75	1.388 (3)
C34—C35	1.384 (3)	C74—H74A	0.9500
C34—H34A	0.9500	C75—C76	1.386 (3)
C35—C36	1.383 (3)	C75—H75A	0.9500
C35—H35A	0.9500	C76—H76A	0.9500
			
N2—C1—N7	99.56 (13)	C34—C35—H35A	120.5
N2—C1—C6	107.62 (14)	C35—C36—C37	120.77 (19)
N7—C1—C6	104.06 (13)	C35—C36—H36A	119.6
N2—C1—H1A	114.7	C37—C36—H36A	119.6
N7—C1—H1A	114.7	C36—C37—C32	120.52 (18)
C6—C1—H1A	114.7	C36—C37—H37A	119.7
C21—N2—C1	119.82 (15)	C32—C37—H37A	119.7
C21—N2—C3	121.33 (15)	C56—C51—C52	118.57 (17)
C1—N2—C3	105.70 (13)	C56—C51—N5	122.19 (16)
N31—C3—N2	110.87 (14)	C52—C51—N5	119.16 (16)
N31—C3—C4	115.18 (15)	C53—C52—C51	120.51 (18)
N2—C3—C4	99.98 (13)	C53—C52—H52A	119.7
N31—C3—H3A	110.1	C51—C52—H52A	119.7
N2—C3—H3A	110.1	C54—C53—C52	120.67 (18)
C4—C3—H3A	110.1	C54—C53—H53A	119.7
N7—C4—N5	104.03 (13)	C52—C53—H53A	119.7
N7—C4—C3	100.84 (13)	C53—C54—C55	119.03 (18)
N5—C4—C3	107.43 (14)	C53—C54—H54A	120.5
N7—C4—H4A	114.4	C55—C54—H54A	120.5
N5—C4—H4A	114.4	C54—C55—C56	120.97 (18)
C3—C4—H4A	114.4	C54—C55—H55A	119.5
C51—N5—C6	122.58 (15)	C56—C55—H55A	119.5
C51—N5—C4	119.89 (14)	C55—C56—C51	120.24 (17)
C6—N5—C4	106.07 (13)	C55—C56—H56A	119.9
N61—C6—N5	108.27 (13)	C51—C56—H56A	119.9
N61—C6—C1	116.89 (15)	C62—N61—C6	123.55 (14)
N5—C6—C1	99.55 (13)	C62—N61—H61N	115.4
N61—C6—H6A	110.5	C6—N61—H61N	109.8
N5—C6—H6A	110.5	N61—C62—C67	123.32 (17)
C1—C6—H6A	110.5	N61—C62—C63	118.01 (16)
C71—N7—C4	119.85 (14)	C67—C62—C63	118.60 (17)
C71—N7—C1	116.75 (14)	C64—C63—C62	120.89 (18)
C4—N7—C1	94.11 (13)	C64—C63—H63A	119.6
C22—C21—C26	118.28 (16)	C62—C63—H63A	119.6
C22—C21—N2	120.50 (16)	C63—C64—C65	120.44 (19)
C26—C21—N2	121.17 (16)	C63—C64—H64A	119.8
C23—C22—C21	120.80 (17)	C65—C64—H64A	119.8
C23—C22—H22A	119.6	C66—C65—C64	118.98 (19)
C21—C22—H22A	119.6	C66—C65—H65A	120.5
C24—C23—C22	120.62 (18)	C64—C65—H65A	120.5
C24—C23—H23A	119.7	C65—C66—C67	121.38 (19)
C22—C23—H23A	119.7	C65—C66—H66A	119.3
C23—C24—C25	118.97 (18)	C67—C66—H66A	119.3
C23—C24—H24A	120.5	C66—C67—C62	119.67 (19)
C25—C24—H24A	120.5	C66—C67—H67A	120.2
C24—C25—C26	120.94 (19)	C62—C67—H67A	120.2
C24—C25—H25A	119.5	C72—C71—C76	119.23 (17)
C26—C25—H25A	119.5	C72—C71—N7	122.79 (17)
C25—C26—C21	120.38 (18)	C76—C71—N7	117.96 (16)
C25—C26—H26A	119.8	C73—C72—C71	119.93 (18)
C21—C26—H26A	119.8	C73—C72—H72A	120.0
C32—N31—C3	119.19 (15)	C71—C72—H72A	120.0
C32—N31—H31N	114.8	C74—C73—C72	120.86 (18)
C3—N31—H31N	109.9	C74—C73—H73A	119.6
C37—C32—C33	118.39 (16)	C72—C73—H73A	119.6
C37—C32—N31	120.28 (17)	C73—C74—C75	119.27 (19)
C33—C32—N31	121.24 (17)	C73—C74—H74A	120.4
C34—C33—C32	120.37 (19)	C75—C74—H74A	120.4
C34—C33—H33A	119.8	C76—C75—C74	120.74 (19)
C32—C33—H33A	119.8	C76—C75—H75A	119.6
C35—C34—C33	121.0 (2)	C74—C75—H75A	119.6
C35—C34—H34A	119.5	C75—C76—C71	119.91 (18)
C33—C34—H34A	119.5	C75—C76—H76A	120.0
C36—C35—C34	118.93 (18)	C71—C76—H76A	120.0
C36—C35—H35A	120.5		
			
N7—C1—N2—C21	179.24 (14)	C3—N31—C32—C37	−137.18 (17)
C6—C1—N2—C21	71.05 (19)	C3—N31—C32—C33	46.4 (2)
N7—C1—N2—C3	37.63 (16)	C37—C32—C33—C34	−0.5 (3)
C6—C1—N2—C3	−70.55 (16)	N31—C32—C33—C34	176.03 (18)
C21—N2—C3—N31	95.41 (19)	C32—C33—C34—C35	0.1 (3)
C1—N2—C3—N31	−123.70 (15)	C33—C34—C35—C36	0.1 (3)
C21—N2—C3—C4	−142.62 (16)	C34—C35—C36—C37	0.1 (3)
C1—N2—C3—C4	−1.74 (17)	C35—C36—C37—C32	−0.6 (3)
N31—C3—C4—N7	83.41 (17)	C33—C32—C37—C36	0.7 (3)
N2—C3—C4—N7	−35.43 (16)	N31—C32—C37—C36	−175.83 (16)
N31—C3—C4—N5	−167.99 (14)	C6—N5—C51—C56	−12.2 (3)
N2—C3—C4—N5	73.16 (16)	C4—N5—C51—C56	−150.44 (16)
N7—C4—N5—C51	179.59 (14)	C6—N5—C51—C52	171.14 (15)
C3—C4—N5—C51	73.24 (19)	C4—N5—C51—C52	32.9 (2)
N7—C4—N5—C6	35.30 (16)	C56—C51—C52—C53	0.4 (3)
C3—C4—N5—C6	−71.05 (16)	N5—C51—C52—C53	177.24 (17)
C51—N5—C6—N61	93.00 (19)	C51—C52—C53—C54	0.8 (3)
C4—N5—C6—N61	−123.91 (15)	C52—C53—C54—C55	−1.7 (3)
C51—N5—C6—C1	−144.42 (16)	C53—C54—C55—C56	1.3 (3)
C4—N5—C6—C1	−1.33 (16)	C54—C55—C56—C51	−0.1 (3)
N2—C1—C6—N61	−171.17 (14)	C52—C51—C56—C55	−0.8 (3)
N7—C1—C6—N61	83.81 (17)	N5—C51—C56—C55	−177.50 (16)
N2—C1—C6—N5	72.62 (15)	N5—C6—N61—C62	−178.74 (16)
N7—C1—C6—N5	−32.40 (16)	C1—C6—N61—C62	70.0 (2)
N5—C4—N7—C71	70.92 (18)	C6—N61—C62—C67	29.8 (3)
C3—C4—N7—C71	−177.85 (14)	C6—N61—C62—C63	−153.34 (17)
N5—C4—N7—C1	−53.25 (15)	N61—C62—C63—C64	−175.32 (18)
C3—C4—N7—C1	57.98 (14)	C67—C62—C63—C64	1.7 (3)
N2—C1—N7—C71	174.81 (14)	C62—C63—C64—C65	−2.4 (3)
C6—C1—N7—C71	−74.18 (17)	C63—C64—C65—C66	1.4 (3)
N2—C1—N7—C4	−58.68 (14)	C64—C65—C66—C67	0.3 (3)
C6—C1—N7—C4	52.34 (15)	C65—C66—C67—C62	−1.0 (3)
C1—N2—C21—C22	27.5 (2)	N61—C62—C67—C66	176.84 (18)
C3—N2—C21—C22	163.02 (15)	C63—C62—C67—C66	0.0 (3)
C1—N2—C21—C26	−155.37 (17)	C4—N7—C71—C72	2.7 (2)
C3—N2—C21—C26	−19.8 (2)	C1—N7—C71—C72	115.13 (19)
C26—C21—C22—C23	0.3 (3)	C4—N7—C71—C76	−178.65 (16)
N2—C21—C22—C23	177.56 (16)	C1—N7—C71—C76	−66.2 (2)
C21—C22—C23—C24	0.7 (3)	C76—C71—C72—C73	2.1 (3)
C22—C23—C24—C25	−0.7 (3)	N7—C71—C72—C73	−179.29 (16)
C23—C24—C25—C26	−0.3 (3)	C71—C72—C73—C74	−0.1 (3)
C24—C25—C26—C21	1.3 (3)	C72—C73—C74—C75	−1.0 (3)
C22—C21—C26—C25	−1.3 (3)	C73—C74—C75—C76	0.2 (3)
N2—C21—C26—C25	−178.49 (16)	C74—C75—C76—C71	1.8 (3)
N2—C3—N31—C32	−169.47 (15)	C72—C71—C76—C75	−2.9 (3)
C4—C3—N31—C32	77.9 (2)	N7—C71—C76—C75	178.37 (17)

### Table 1 Geometric parameters relating anomeric interactions in N'—C—N" fragments (Å, °)

**Table:**

Parameters	*n*_N31_→σ*_C3—N2_	*n*_N2_→σ*_C2—N7_	*n*_N7_→σ*_C4—N5_	*n*_N61_→σ*_C6—N5_
N'—C	1.454 (2)	1.469 (2)	1.451 (2)	1.451 (2)
C—N''	1.472 (2)	1.472 (2)	1.480 (2)	1.466 (2)
N'—C—N''	110.87 (14)	99.56 (13)	104.03 (13)	108.27 (13)
Pyr N'^a,b^	343.89	346.85 (25)	330.71 (19)	348.75
Pyr N''	346.85 (25)	330.71 (19)	348.54 (24)	348.54 (24)

Notes: (a) Pyr denotes the pyramidality of the N atoms, the sum of the three angles around the N atom. (b) s.u. values are estimated from the sum of s.u. values when they are available.
